# Glycoconjugates tune the viscoelastic signature of mammary tumor progression

**DOI:** 10.64898/2026.10.20.760544

**Published:** 2026-10-21

**Authors:** Abhishek Sharma, Jane A. Baude, Maya Papez, Marissa A. Gionet-Gonzales, Ryan S. Stowers

## Abstract

Breast cancer progression involves extensive remodeling of the tumor extracellular matrix (ECM), resulting in changes in mechanical properties. Tumors are viscoelastic, exhibiting both an initial elastic response to loading and a time-dependent viscous response. While it is well established that tumor elasticity or stiffness is elevated, changes in tissue viscoelasticity during breast cancer progression remain poorly characterized. Here, using a mouse model that recapitulates stages of human breast cancer progression (mouse mammary tumor virus-polyoma middle T antigen, MMTV-PyMT), we show that tumors from both early and late disease time points were significantly more viscoelastic than normal mammary glands. Spatial microindentation revealed that while local stiffness and viscoelasticity are strongly coupled in soft regions of normal tissue, this relationship weakens in late carcinoma. Compared to normal glands, carcinoma tissues also exhibited marked upregulation of glycosaminoglycans (GAGs) and glycoproteins. Selective enzymatic degradation of sulfated GAGs, mucins, or O/N-linked glycans reduced viscoelasticity without altering stiffness, establishing glycoconjugates as major contributors to tumor viscoelasticity. In vitro, increasing matrix viscoelasticity drove non-transformed mammary epithelial cells (MCF10A) toward a malignant phenotype through focal adhesion kinase mechanosignaling. Our work establishes increased viscoelasticity as a defining mechanical feature of breast cancer progression, driven in part by the accumulation of glycoconjugates. These findings provide a fuller picture of tumor mechanical evolution and a benchmark for recapitulating matrix viscoelasticity in engineered models of breast cancer.

